# Protein phosphatase 2a (PP2A) binds within the oligomerization domain of striatin and regulates the phosphorylation and activation of the mammalian Ste20-Like kinase Mst3

**DOI:** 10.1186/1471-2091-12-54

**Published:** 2011-10-10

**Authors:** Johnthan Gordon, Juyeon Hwang, Karma J Carrier, Candace A Jones, Quiana L Kern, Carlos S Moreno, Richard H Karas, David C Pallas

**Affiliations:** 1 Department of Biochemistry and Winship Cancer Institute, Emory University School of Medicine, Atlanta, Georgia 30322, USA; 2 Postdoctoral Fellowships in Research & Science Teaching (FIRST) Program, Emory University School of Medicine, Atlanta, Georgia 30322, USA; 3 Biochemistry, Cell, Developmental Biology Graduate Program, Emory University School of Medicine, Atlanta, Georgia 30322, USA; 4 Department of Medicine, Molecular Cardiology Research Institute, Tufts Medical Center, 800 Washington Street, Boston, MA 02111, USA; 5 Current address: Meso Scale Discovery, 9238 Gaither Rd, Gaithersburg, MD 20877, USA; 6 Current address: North Louisiana Criminalistics Laboratory, 1115 Brooks Street, Shreveport, LA 71101, USA; 7 Current address: Department of Anesthesiology, Emory University School of Medicine, Atlanta, GA 30322, USA; 8 Current address: Department of Pathology and Laboratory of Medicine, Emory University School of Medicine, Atlanta, GA 30322, USA

## Abstract

**Background:**

Striatin, a putative protein phosphatase 2A (PP2A) B-type regulatory subunit, is a multi-domain scaffolding protein that has recently been linked to several diseases including cerebral cavernous malformation (CCM), which causes symptoms ranging from headaches to stroke. Striatin association with the PP2A A/C (structural subunit/catalytic subunit) heterodimer alters PP2A substrate specificity, but targets and roles of striatin-associated PP2A are not known. In addition to binding the PP2A A/C heterodimer to form a PP2A holoenzyme, striatin associates with cerebral cavernous malformation 3 (CCM3) protein, the mammalian Mps one binder (MOB) homolog, Mob3/phocein, the mammalian sterile 20-like (Mst) kinases, Mst3, Mst4 and STK25, and several other proteins to form a large signaling complex. Little is known about the molecular architecture of the striatin complex and the regulation of these sterile 20-like kinases.

**Results:**

To help define the molecular organization of striatin complexes and to determine whether Mst3 might be negatively regulated by striatin-associated PP2A, a structure-function analysis of striatin was performed. Two distinct regions of striatin are capable of stably binding directly or indirectly to Mob3--one N-terminal, including the coiled-coil domain, and another more C-terminal, including the WD-repeat domain. In addition, striatin residues 191-344 contain determinants necessary for efficient association of Mst3, Mst4, and CCM3. PP2A associates with the coiled-coil domain of striatin, but unlike Mob3 and Mst3, its binding appears to require striatin oligomerization. Deletion of the caveolin-binding domain on striatin abolishes striatin family oligomerization and PP2A binding. Point mutations in striatin that disrupt PP2A association cause hyperphosphorylation and activation of striatin-associated Mst3.

**Conclusions:**

Striatin orchestrates the regulation of Mst3 by PP2A. It binds Mst3 likely as a dimer with CCM3 via residues lying between striatin's calmodulin-binding and WD-domains and recruits the PP2A A/C heterodimer to its coiled-coil/oligomerization domain. Residues outside the previously reported coiled-coil domain of striatin are necessary for its oligomerization. Striatin-associated PP2A is critical for Mst3 dephosphorylation and inactivation. Upon inhibition of PP2A, Mst3 activation appears to involve autophosphorylation of multiple activation loop phosphorylation sites. Mob3 can associate with striatin sequences C-terminal to the Mst3 binding site but also with sequences proximal to striatin-associated PP2A, consistent with a possible role for Mob 3 in the regulation of Mst3 by PP2A.

## Background

Protein phosphatase 2A (PP2A) is a multifunctional serine/threonine phosphatase found in all eukaryotes that regulates a host of cellular processes [1,2]. It is composed of a core heterodimer consisting of a structural A subunit and a catalytic C subunit [1], which further complexes with regulatory B-type subunits to form heterotrimeric PP2A holoenzymes. The B-type subunits direct PP2A to specific signaling complexes and subcellular locations [1] and regulate the activity of the C subunit towards different substrates (for examples, see [3-8]). While there are only two isoforms of the A and C subunits (α, β), there are several families of B-type subunits (B55/B, B56/B', and B''). In addition to these three families of B-type subunits, the striatin family of scaffolding proteins (striatin, S/G_2 _nuclear autoantigen (SG2NA), and zinedin) represents a possible fourth family of PP2A B-type subunits in that they bind PP2A A/C heterodimers in the absence of other B-type subunits [8,9] and alter the activity of the bound PP2A A/C heterodimer [8].

**Figure 1:**
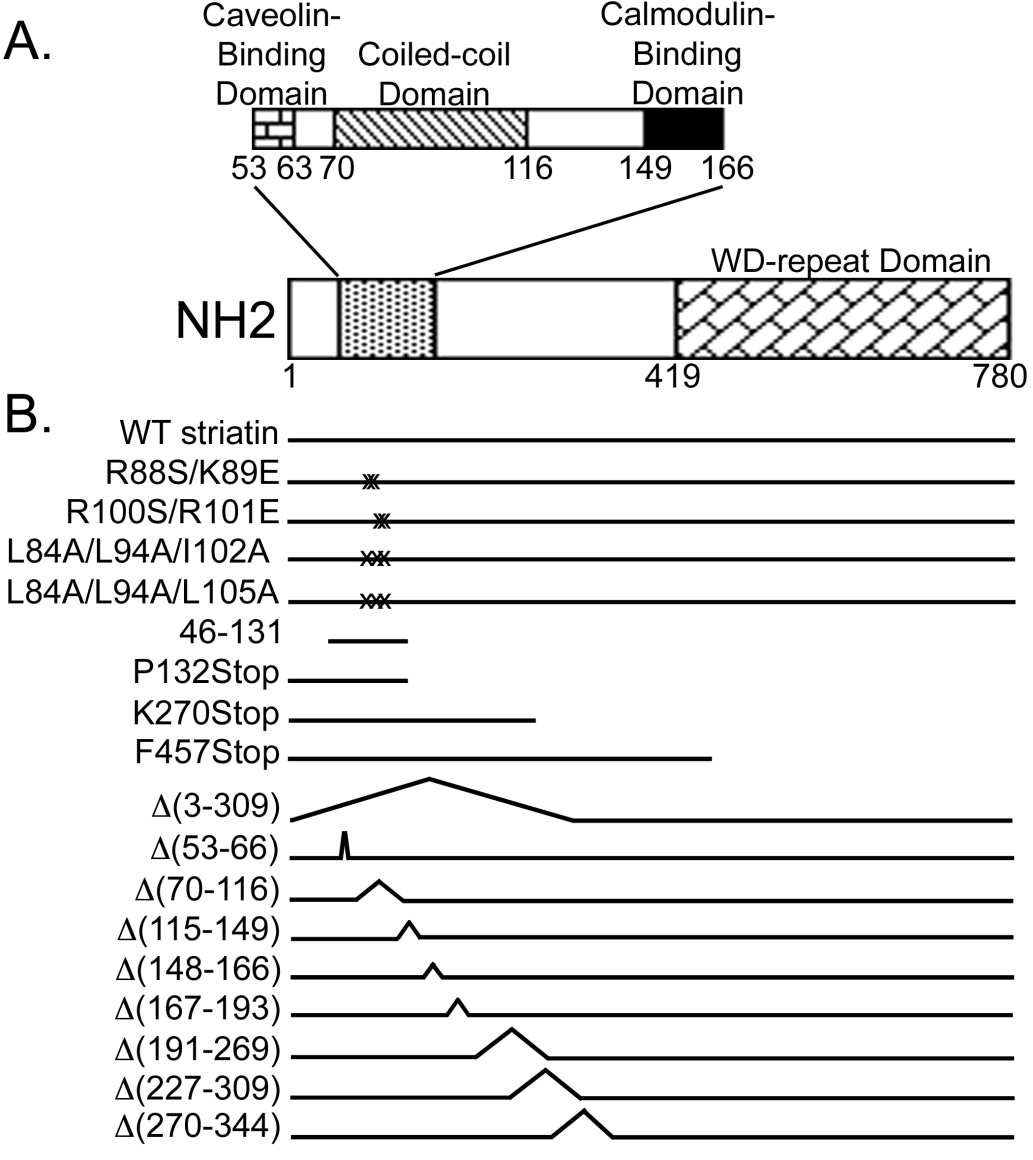
**Schematic of the structures of human wild-type and mutant striatin proteins**. **(A) **Schematic of wild-type striatin, drawn to scale, showing the locations of its previously published protein-interaction domains. White boxes represent regions of unknown function. **(B) **Stick diagrams of wild-type and mutant striatins used in this study. Point mutations are denoted by X's, and deletions are shown as peaks in the stick diagrams or absence of a line. Positions of mutants are noted relative to wild-type striatin domains in panel A.

Striatin family members serve as molecular scaffolds that organize large signaling complexes. Striatin, SG2NA, and zinedin contain multiple protein-binding domains: a caveolin-binding domain [10], a coiled-coil domain [11], a Ca^2+^-calmodulin-binding domain [12], and a WD-repeat domain [13] (Figure 1A). Consistent with the presence of these domains, striatin family members have been reported to associate with calmodulin in a Ca^2+^-dependent manner [8,11,13] and with caveolin-1 [10]. In addition, striatin family members oligomerize with each other [14] and the coiled-coil domain has been shown to mediate this interaction [15]. Other proteins found in complexes with striatin family members include, but are not limited to, Mob3/phocein (called Mob3 herein) [14,16], which is involved in vesicular trafficking [16-18]; Mst3, Mst4, and STK25 [9], members of the Germinal Center Kinase-III (GCK-III) subfamily of sterile 20-like kinases recently implicated in control of cell migration, cell cycle, Golgi assembly, and cell polarity [19-22]; cerebral cavernous malformation 3 (CCM3) protein [9], which is required for stabilization of the GCK-III kinases and thus for their function [21]; and striatin-interacting proteins (STRIP) 1 and 2 [9]. In addition, striatin family complexes containing PP2A, Mob3, GCK-III kinases, CCM3, and STRIP 1 and 2 can also associate mutually exclusively with additional components [9]. Finally, striatin has been found to bind Gαi and estrogen receptor α, facilitating rapid non-genomic signaling of estrogen receptor α [23].

Several lines of evidence suggest that a common function of striatin family complexes is their involvement in endocytosis and vesicular trafficking. Striatin, SG2NA and the common associated protein Mob3 are localized to the Golgi, cytoplasm, and plasma membrane [14,16] and the Golgi localizations of SG2NA and Mob3 are rapidly altered by exposure of cells to brefeldin A, an inhibitor of Arfs, small G proteins known to regulate vesicular trafficking [16]. In addition, striatin, SG2NA, and Mob3 all associate with proteins implicated in endocytosis and vesicular trafficking [17,24]. Finally, both CCM3 and the Drosophila Mob3/phocein homolog, DMob4, have been directly implicated in endocytosis and/or vesicular trafficking [18,25].

Striatin complexes have been linked to several clinical conditions. The CCM3 protein found in striatin complexes is one of three gene products mutated in cerebral cavernous malformation, a common type of angioma [9,26-28]. Moreover, a small deletion in the 3' untranslated region of striatin that leads to lower levels of striatin mRNA was recently implicated in a canine model of arrhythmogenic right ventricular cardiomyopathy [29]. Finally, the striatin gene was also found in one of twenty-two loci containing common variants associated with QRS interval length and cardiac ventricular conduction [30]. Thus, the study of striatin family complexes is of great interest because of their potential roles in vesicular trafficking, Golgi assembly, cell polarity, cell migration, cell cycle, signaling, and disease.

Despite the identification of a large number of components of striatin family complexes, little is known about the architecture of these complexes and their regulation. Defining the molecular organization of striatin family complexes is critical to understanding how they function. Equally important is the elucidation of the targets, roles, and regulation of the phosphatase and kinase enzymes in these complexes. Striatin association with the PP2A A/C heterodimer alters PP2A's substrate specificity [8], but the targets and role(s) of striatin-associated PP2A are not known. Treatment of cells with okadaic acid at concentrations known to inhibit PP2A induces hyperphosphorylation of striatin, SG2NA, Mob3, and other unidentified striatin family binding partners, including proteins that migrate at the size and isoelectric point of Mst3 and Mst4 [14]. Thus, striatin-associated PP2A may regulate the function of striatin-family complexes by modulating the phosphorylation state of striatin and its associated proteins, including the Mst3 and Mst4 kinases. However, this hypothesis has not been directly tested.

To help define the molecular organization of striatin complexes and to determine whether Mst3 might be regulated by striatin-associated PP2A, we performed a structure-function analysis of striatin, defining domains of striatin important for the binding of a number of striatin binding partners and determining the effect of PP2A-deficient striatin mutants on the activation state of Mst3. To gain insight into the mechanism of Mst3 activation by autophosphorylation, we also performed a mutational analysis of Mst3 activation loop phosphorylation sites. Our results greatly enhance our understanding of the architecture of striatin family complexes, uncover unique binding requirements for PP2A, and provide new insight into the oligomerization of striatin family members and into the mechanism of activation of Mst3 by autophosphorylation. Overall, they support a model in which striatin orchestrates the regulation of Mst3 by PP2A to regulate Mst3 function in the cell.

## Results

### Generation of striatin mutants for structure-function analysis of striatin complexes

To facilitate dissection of the molecular organization of striatin complexes, we generated a set of deletion mutants and a complementary set of point mutants of striatin for use in our experiments. Because all cells tested to date contain endogenous striatin, all striatin mutants were constructed with an N-terminal Hemagglutinin (HA)-epitope tag to allow specific immunoprecipitation and detection of the exogenously expressed wild-type and mutant striatins. Figure 1A shows the known domain organization of striatin while Figure 1B shows a schematic of the different striatin mutants used in this study on the same scale as Figure 1A.

### The WD-repeats of striatin are not required for PP2A binding but contribute to Mob3 association

**Figure 2:**
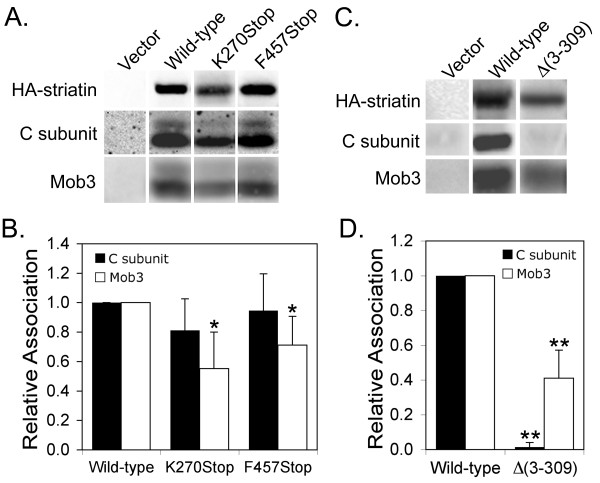
**The N-terminal region of striatin binds PP2A while Mob3 associates with both N-terminal and more C-terminal striatin sequences**. **(A) **HEK293 cells transfected with HA-tagged wild-type and mutant striatins were lysed and HA-striatin immune complexes were isolated, separated by SDS-PAGE, and proteins contained in the immunoprecipitates were detected by immunoblotting using antibodies that recognize the HA-epitope tag, PP2A C subunit, and Mob3. **(B) **Relative binding of PP2A C subunit and Mob3 to wild-type striatin and C-terminal truncation mutants of striatin was determined by quantitatively comparing the C subunit/HA-striatin and Mob3/HA-striatin ratios of wild-type striatin to those of the mutants using a Bio-Rad Fluor S-Max chemilumimager (see Methods). **(C) **Immunoblot showing that Mob3 also associates with residues 310-780 of striatin while PP2A does not. **(D) **Relative binding of PP2A C subunit and Mob3 to wild-type and Δ(3-309) striatins was measured in the same manner as described in *B*. The HA-striatins migrated at different positions due to their size differences but are shown side by side in panels A and C for comparison of their levels. C subunit can migrate as either singlets or doublets; whether double or single bands are seen can vary for the same sample from gel to gel. This pattern of migration in SDS-PAGE has been noted previously for endogenous PP2A C subunits [40-43] and is not due to degradation. The error bars in panels B and D represent the standard deviation of at least three independent experiments. *, p ≤ 0.05; **, p ≤ 0.01 relative to wild-type.

The A and C subunits of PP2A are known to bind striatin [8] but the region of striatin that binds to PP2A is unknown. As a first approach to identifying striatin sequences important for PP2A association, we compared the relative ability of HA-epitope tagged wild-type striatin and two HA-tagged striatin C-terminal deletion mutants, K270Stop and F457Stop (Figure 1), to bind PP2A in vivo. Both bind PP2A (Figure 2A). Quantitation of the ratio of PP2A bound to striatin shows that loss of seven of eight of striatin's WD-repeats (F457Stop) has no effect on PP2A association while loss of all eight WD-repeats plus the central region of striatin (K270Stop) has only a small effect on PP2A binding that was not statistically significant (Figure 2B). These data indicate that residues 270-780 of human striatin are largely dispensable for PP2A association and that the first 269 amino acids of striatin contain the primary PP2A binding domain.

To determine the importance of C-terminal striatin sequences for Mob3/striatin complex formation, the same immunoprecipitates were probed for Mob3. Mob3, like PP2A, also binds to both of the C-terminal deletion mutants (Figure 2A). Thus, amino acids 1-269 in striatin also contain a domain that binds Mob3, either directly or indirectly. However, quantitation of results from multiple experiments shows that Mob3 binds at reduced levels to both of the C-terminal mutants (Figure 2B). Loss of seven WD-repeats (F457Stop) or all eight WD-repeats plus the central region of striatin (K270Stop) results in a 29% or 45% reduction in Mob3 binding, respectively. These results demonstrate that the WD-repeats are important for efficient association of Mob3 and that residues between 270 and 457 may also contribute. Thus, striatin residues 1-269 contain binding sites for both Mob3 and PP2A.

### Both N- and C-terminal sequences of striatin associate with Mob3 but only N-terminal striatin sequences associate stably with PP2A

The reduced binding of Mob3 to both of the striatin C-terminal deletion mutants suggested that striatin sequences beyond residue 269 might bind Mob3. To test for this possibility, immunoprecipitates of Δ(3-309) striatin, an N-terminal deletion mutant of striatin (Figure 1), were probed for the presence of Mob3. The Δ(3-309) striatin mutant bound Mob3 at approximately 40% the level of wild-type striatin (Figure 2C-D). Thus, there are at least two distinct domains within striatin (aa1-269 and aa310-780) capable of interacting directly or indirectly with Mob3. PP2A C subunit, on the other hand, did not bind to Δ(3-309) striatin (Figure 2C-D), demonstrating that PP2A associates stably only with residues located in the N-terminal region of striatin.

### The coiled-coil and caveolin-binding domains of striatin, but not the calmodulin-binding domain of striatin, are necessary for oligomerization and for PP2A binding

**Figure 3:**
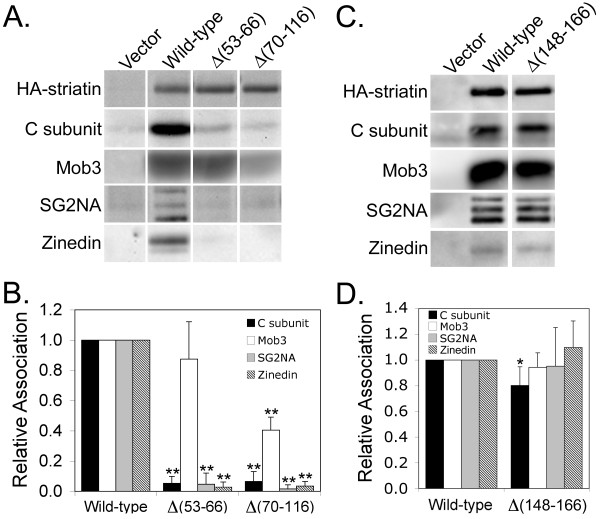
**The coiled-coil and caveolin-binding domains are required for oligomerization and PP2A binding but the calmodulin-binding domain is not**. **(A) **HEK293 cells transfected with HA-tagged wild-type and mutant striatins were lysed and HA-striatin immune complexes were isolated, separated by SDS-PAGE, and proteins contained in the immunoprecipitates were detected by immunoblotting using antibodies that recognize the HA-epitope tag, PP2A C subunit, Mob3, SG2NA, and zinedin. Deletion of either the coiled-coil domain [Δ(70-116) striatin] or the caveolin-binding domain [Δ(53-66) striatin] of striatin resulted in loss of oligomerization and PP2A binding. **(B) **Relative binding of wild-type and deletion mutant striatins to PP2A C subunit, Mob3, SG2NA, and zinedin was measured as described in the legend to Figure 2B. **(C) **A similar experiment to (A) was conducted with wild-type striatin and Δ(148-166) striatin, which deletes the striatin calmodulin-binding motif. **(D) **Relative binding of wild-type and mutant striatins to PP2A C subunit, Mob3, SG2NA, and zinedin was measured as described in the legend to Figure 2B. The HA-striatins migrated at different positions due to their size differences but are shown side by side in panels A and C for comparison of their levels. The error bars in panels B and D represent the standard deviation of at least three independent experiments. *, p ≤ 0.05; **, p ≤ 0.01 relative to wild-type.

To further localize the PP2A-binding domain within the N-terminal 269 amino acids of striatin, HA-tagged striatin mutants lacking previously identified N-terminal protein-interaction domains (caveolin-binding, coiled-coil, or calmodulin-binding) were created (Figure 1). Co-immunoprecipitation was utilized to test the abilities of these mutants to bind PP2A C subunit (Figure 3). The coiled-coil domain of striatin is required for binding PP2A, since a coiled-coil deletion mutant, Δ(70-116), is unable to specifically co-precipitate PP2A C subunit (Figure 3A-B). It has been reported that the coiled-coil domain is the oligomerization domain of the striatin family of proteins [15]. In agreement with this, Δ(70-116) striatin fails to oligomerize with SG2NA and zinedin, as demonstrated by the absence of SG2NA and zinedin in immunoprecipitates of this mutant (Figure 3A-B). Δ(70-116) also shows reduced ability to bind Mob3, binding only 40% of wild-type levels. Considering the fact that there is a Mob3 binding domain in aa1-269 of striatin that can bind approximately 55% of the wild-type level of Mob3 (Figure 2B), these results suggest that the coiled-coil domain contains binding sites for both PP2A and Mob3.

In addition to the coiled-coil domain, striatin has been reported to bind caveolin-1 through a caveolin-binding domain found within amino acid residues 53-63 [10]. To determine the role, if any, of the caveolin-binding domain in striatin association with PP2A and Mob3, a mutant of striatin lacking the caveolin-binding domain, Δ(53-66) striatin, was analyzed. Co-immunoprecipitation studies revealed that Δ(53-66) striatin binds little to no PP2A (Figure 3A-B). In contrast, the Δ(53-66) striatin mutant could co-precipitate Mob3 at near wild-type levels, confirming that loss of PP2A binding is specific and not due to protein misfolding. Interestingly, this mutant is completely defective in oligomerization as indicated by its inability to bind SG2NA and zinedin (Figure 3A-B), indicating that one or more residues within 53-66 are essential for striatin oligomerization.

Finally, we examined the importance of the calmodulin-binding domain in the N-terminus of striatin by analyzing the striatin deletion mutant, Δ(148-166), which deletes this entire domain (Figure 1). Loss of striatin's calmodulin-binding domain has only a small effect on PP2A C subunit binding, and no significant effect on Mob3 binding or oligomerization with SG2NA and zinedin (Figure 3C-D).

### Residues in the coiled-coil domain of striatin are critical for PP2A C subunit association independent from their role in oligomerization

**Figure 4:**
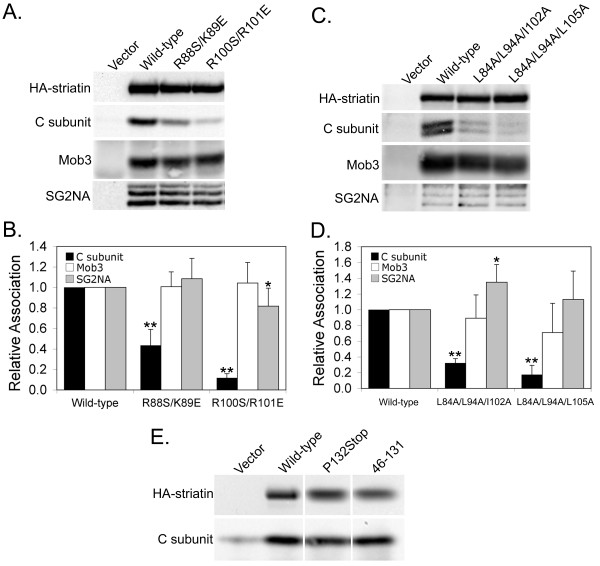
**Conserved residues within the coiled-coil domain of striatin are critical for association with PP2A but not with Mob3**. **(A) **HEK293 cells transfected with HA-tagged wild-type and coiled-coil mutant striatins were lysed and HA-striatin immune complexes were isolated and proteins contained in the immunoprecipitates were detected by immunoblotting using antibodies that recognize the HA-epitope tag, PP2A C subunit, Mob3, and SG2NA. **(B) **Relative binding of wild-type and point mutant striatins to PP2A C subunit, Mob3, and SG2NA was measured as described in the legend to Figure 2B. The error bars represent the standard deviation of at least three independent experiments. *, p ≤ 0.05; **, p ≤ 0.01 relative to wild-type. **(C) **A similar experiment to (A) was conducted with wild-type striatin and striatin mutants with hydrophobic coiled-coil residue substitutions. **(D) **Relative binding of wild-type and point mutant striatins to PP2A C subunit, Mob3, and SG2NA was measured as described in the legend to Figure 2B. The error bars represent the standard deviation of at least three independent experiments. *, p ≤ 0.05; **, p ≤ 0.01 relative to wild-type. The difference in C subunit association between L84A/L94A/I102A and L84A/L94A/L105A was also significant (p = 0.029). **(E) **A similar experiment to (A) was conducted with wild-type striatin and small, coiled-coil-containing mutants, P132Stop striatin and 46-131 striatin. The HA-striatins migrated at different positions but are shown side by side for comparison of their levels. Although some non-specific sticking of C subunit was seen in the vector control lane, both mutants bound wild-type levels of PP2A C subunit.

The fact that the coiled-coil domain of striatin is required for its association with PP2A suggested that PP2A might bind directly to this region. If so, the introduction of point mutants in the coiled-coil domain may disrupt PP2A binding without interfering with striatin oligomerization. To test this hypothesis, we compared the peptide sequence of the coiled-coil domain of striatin from several species and identified residues that were completely conserved. Four such residues were Arg^88^, Lys^89^, Arg^100 ^and Arg^101^. Pairs of these residues were substituted with uncharged serine residues or residues of opposite charge in an attempt to disrupt striatin/PP2A association. The resulting double point mutants, R88S/K89E and R100S/R101E striatin, were then analyzed for PP2A, Mob3, and SG2NA binding. The mutants R88S/K89E and R100S/R101E are able to bind only 40% and 10% of wild-type levels of PP2A C subunit, respectively (Figure 4A-B). Binding of Mob3 was not reduced. Both mutants retained the ability to efficiently bind SG2NA at wild-type (R88S/K89E) or near wild-type (81%; R100S/R101E) levels (Figure 4A-B), indicating that effects on PP2A C subunit can be separated from effects on oligomerization. These results support the hypothesis that PP2A binds directly to the coiled-coil domain of striatin.

To further investigate PP2A binding in the coiled-coil domain, four conserved hydrophobic residues (leucines 84, 94, and 105, and isoleucine 102) were replaced with alanine. Two triple mutants (L84A/L94A/I102A and L84A/L94A/L105A) reduced PP2A binding to 32% to 17% of wild-type levels, while striatin oligomerization remained intact (Figure 4C-D). No statistically significant reduction in the binding of Mob3 was seen. Together, these results show that critical residues in the PP2A binding domain of striatin are within the central portion of the coiled-coil domain between residues 84 and 105.

To define whether the coiled-coil domain was sufficient to bind PP2A and to test the importance of the amino acids preceding the caveolin-binding domain for PP2A binding, two additional mutants (P132Stop and 46-131) were constructed (Figure 1). Both of these mutants contain the coiled-coil domain. Because the caveolin-binding domain is also required for PP2A binding (Figure 3) both mutants were designed to retain this domain as well, but mutant 46-131 lacks most of the amino acids that precede it. PP2A binds efficiently to both mutants, demonstrating that the binding site for PP2A is within striatin amino acids 46-131 and that the first forty-five amino acids of striatin are dispensable for PP2A binding (Figure 4E).

### Determinants within striatin residues 191-344 are critical for binding to Mst3 and Mst4 kinases and to CCM3

**Figure 5:**
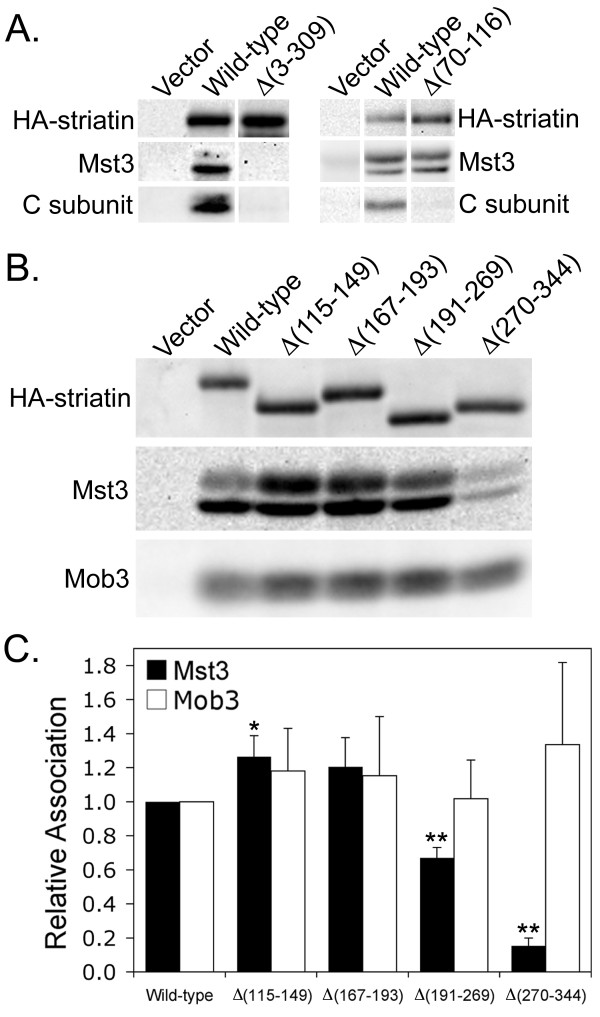
**Characterization of Mst3 association with striatin**. **(A) **Three days after transfection with HA-tagged wild-type and mutant striatins, HEK293 cells were lysed and HA-striatin immune complexes were isolated, separated by SDS-PAGE, and immunoblotted. Representative immunoblots are shown. *Left*, Mst3 binding, like PP2A C subunit binding, requires N-terminal sequences of striatin. *Right*, unlike PP2A C subunit, Mst3 binding does not require the striatin coiled-coil domain or striatin oligomerization. **(B) **A similar experiment to (A) was conducted to compare the abilities of Mst3 and Mob3 to bind wild-type striatin and a set of internal striatin deletion mutants localized to regions of unknown function. **(C) **Relative binding of wild-type and mutant striatins to Mst3 and Mob3 was measured as described in the legend to Figure 2B. The error bars represent the standard deviation of at least three independent experiments. *, p ≤ 0.05; **, p ≤ 0.01 relative to wild-type.

We next investigated the association of striatin with Mst3 kinase, a recently discovered component of striatin family complexes [9]. Initial experiments showed that Mst3 does not bind to Δ(3-309) striatin (Figure 5A), indicating that striatin residues before 310 are critical for its association. However, Mst3 bound well to Δ(70-116) striatin (Figure 5A) and Δ(53-66) (data not shown), indicating that Mst3 association with striatin does not require the coiled-coil or caveolin-binding domains or oligomerization of striatin. This result also indicates that the loss of PP2A binding to Δ(70-116) seen in Figure 3A-B is not simply due to misfolding of this mutant.

To further delineate the striatin residues needed for Mst3 binding, the ability of Mst3 to associate with a set of deletion mutants spanning regions of unknown function between the coiled-coil domain and residue 344 of striatin (Figure 1) was measured. The results of this analysis (Figure 5B-C) indicate that deletion of the residues between the coiled-coil domain and the calmodulin domain and deletions after the calmodulin-binding domain up to residue 193 cause no reduction in Mst3 binding. However, deletion of residues 191-269 causes a ~33% reduction in Mst3 binding while loss of residues 270-344 causes an ~85% loss of Mst3 binding. Deletion of striatin amino acids 191-269 and 270-344 also reduced Mst4 binding by 33 ± 5% and 75 ± 3%, respectively (average ± range of two experiments). These results indicate that the Mst3 and Mst4 binding sites probably span across amino acid 269, with the strongest interactions being C-terminal to this residue.

**Figure 6:**
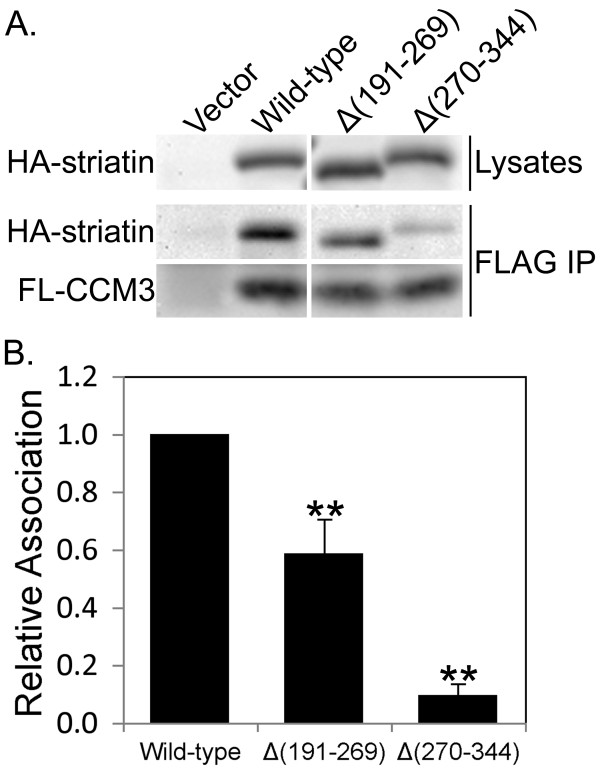
**CCM3 binding to striatin is affected by the same deletions that reduce Mst3 binding**. **(A) **Three days after transfection with HA-tagged wild-type and mutant striatins, HEK293 cells stably expressing FLAG-CCM3 were lysed and FLAG-CCM3 immune complexes were isolated, separated by SDS-PAGE, and immunoblotted. Lysate levels of the transfected HA-tagged striatins are shown for comparison to the amount of HA-striatins that coimmunoprecipitated with FLAG-CCM3. **(B) **Relative binding of wild-type and mutant striatins to FLAG-CCM3 was measured as described in the legend to Figure 2B, except that in this experiment the values were normalized to expression of the HA-constructs in the cell lysates. The error bars represent the standard deviation of three independent experiments. **, p ≤ 0.01 relative to wild-type.

Because CCM3 has been reported to bind and stabilize Mst3 and Mst4 [21], the importance of striatin residues 191-269 and 270-344 for CCM3 binding was tested. Since commercially available CCM3 antibodies did not consistently detect CCM3, we established a stable FLAG-tagged CCM3-expressing cell line as described in Methods and analyzed the ability of this CCM3 to associate with HA-tagged wild-type and mutant striatins by co-immunoprecipitation. Deletion of striatin residues 191-269 and 270-344 reduced CCM3 binding ~40% and ~90%, respectively (Figure 6A-B), suggesting that CCM3 and the Mst3 and Mst4 kinases may bind as a complex to this region of striatin.

### The calmodulin-binding domain of striatin negatively regulates association with the Mst3 and Mst4 kinases

**Figure 7:**
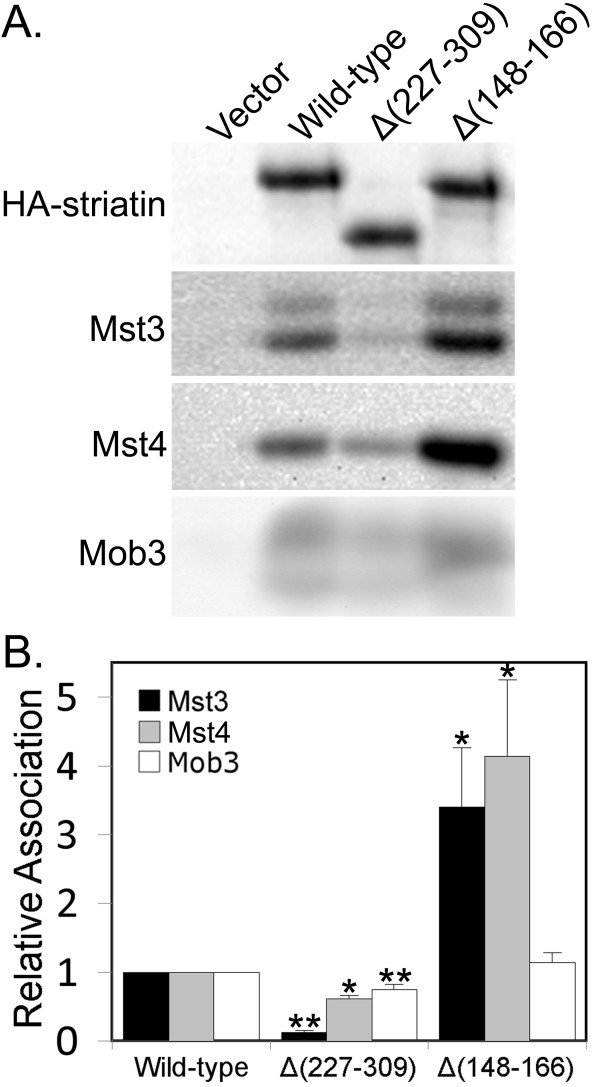
**Loss of striatin residues 227-309 nearly abolishes Mst3 binding while loss of the calmodulin domain greatly enhances the binding of both Mst3 and Mst4**. **(A) **Three days after transfection with HA-tagged wild-type and mutant striatins, HEK293 cells were lysed and HA-striatin immune complexes were isolated, separated by SDS-PAGE, and immunoblotted. The Mob3 panel shown has been compressed 25% in the vertical dimension to save space. **(B) **Relative binding of wild-type and mutant striatins to Mst3, Mst4, and Mob3 was measured as described in the legend to Figure 2B. The error bars represent the standard error of four independent experiments. *, p ≤ 0.05; **, p ≤ 0.01 relative to wild-type.

The deletion mutant, Δ(115-149) striatin, had a statistically significant ~25% increase in the amount of Mst3 bound (Figure 5C). Since this region is just before the calmodulin-binding domain of striatin, we tested the ability of Mst3 to bind to Δ(148-166) striatin, in which the calmodulin-binding domain has been deleted. At the same time, we tested an additional mutant, Δ(227-309) striatin, spanning the region that seemed to affect Mst3 binding the most. The results indicate that Δ(227-309) striatin is almost completely defective in Mst3 binding, while still binding Mob3 at 75% of wild-type levels (Figure 7A-B). In striking contrast, Mst3 bound ~3.5-fold better when the calmodulin-binding domain of striatin was deleted (Figure 7A-B). Interestingly, probing of the same immunoprecipitates for the related striatin-associated kinase, Mst4, revealed a less dramatic reduction (38%) in Mst4 binding to Δ(227-309) striatin but a similar ~4-fold increase in binding to the calmodulin-binding domain deletion mutant.

### Striatin-associated PP2A negatively regulates the phosphorylation of Mst3 kinase

**Figure 8:**
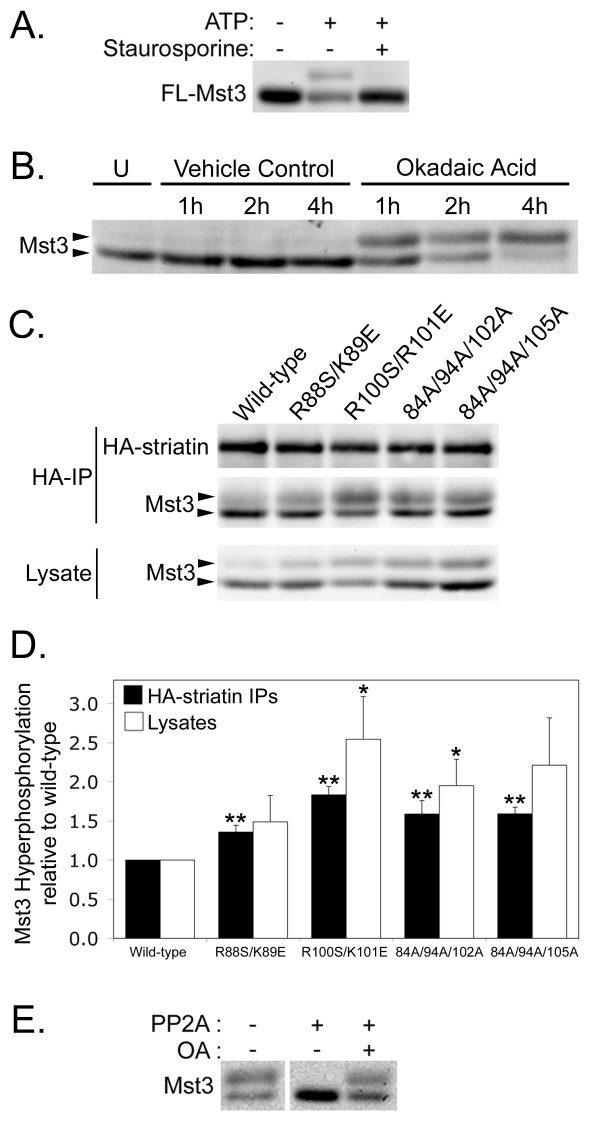
**Loss of PP2A binding to striatin causes Mst3 hyperphosphorylation**. **(A) **FLAG-Mst3 (FL-Mst3) immunoprecipitates were prepared, and then aliquots were incubated without ATP, with ATP, or with ATP plus the kinase inhibitor, staurosporine. FLAG-Mst3 was visualized by immunoblotting. **(B) **Mst3 hyperphosphorylation caused by incubation of HEK293 cells with 100 nM okadaic acid for the times indicated retards the migration of Mst3 in SDS-PAGE. U, untreated. Vehicle Control, DMSO. **(C) **Three days after transfection of HEK293 cells with HA-tagged wild-type striatin and striatin mutants deficient in PP2A binding, HA-striatin immune complexes (HA-IP) and lysates were prepared and immunoblotted. **(D) **Relative hyperphosphorylation of Mst3 in striatin complexes and lysates of transfected cells was quantitated by measuring the ratios of the upper and lower bands of Mst3 using a chemilumimager and normalizing to wild-type HA-striatin. The error bars represent the standard error of at least four independent experiments. *, p ≤ 0.05; **, p ≤ 0.01 relative to wild-type. The p values for R88S/K89E and 84A/94A/105A mutant effects in lysates were 0.19 and 0.06, respectively. **(E) **Three days after transfection of HEK293 cells with HA-tagged R100S/R101E striatin, HA-striatin immune complexes were prepared in the absence of phosphatase inhibitors, denatured, and divided into three equal portions. Samples were incubated without PP2A or with purified PP2A plus either DMSO (vehicle control) or 100 nM okadaic acid. After incubation, the Mst3 protein bands were detected by immunoblotting. All lanes are from the same gel and exposure but the first two lanes were originally separated by a blank lane.

Previously, two-dimensional analysis of SG2NA complexes from ^32^P-inorganic phosphate-labeled cells revealed an ~52 kDa unknown protein whose phosphorylation increased dramatically upon treatment of cells with okadaic acid at concentrations known to inhibit PP2A [14]. The size and estimated isoelectric point of that phosphoprotein are similar to Mst3, raising the possibility that striatin-associated PP2A regulates the phosphorylation state of striatin-associated Mst3. To test this possibility, we took advantage of the fact that phosphorylation slows the mobility of many proteins. Figure 8A shows that upon incubation with ATP and manganese in vitro, a portion of Mst3 undergoes a gel shift on SDS-polyacrylamide gel electrophoresis (SDS-PAGE). The gel shift does not occur in the absence of ATP or if the kinase inhibitor staurosporine is included (Figure 8A), indicating that the shift occurs due to autophosphorylation of Mst3 or phosphorylation of Mst3 by a tightly associated kinase.

Mst3 in lysates from untreated human 293 cells exists predominantly as one major band and a minor, more slowly migrating, upper band (Figure 8B, first lane). Upon treatment with 100 nM okadaic acid, the Mst3 in the lower band shifts to the upper band over time until at 4 h the upper band becomes the predominant band (Figure 8B). It was previously shown that treatment of mammalian cells with 100 nM okadaic acid for 6 h-24 h inhibits most cellular PP2A without significantly inhibiting PP1 because of the slow rate at which okadaic acid enters cells [31]. Considered together, these results indicate that Mst3 phosphorylation may be regulated by PP2A.

Okadaic acid-induced phosphorylation of Mst3 could be due to inhibition of PP2A in the striatin complex or to inhibition of another PP2A holoenzyme responsible for regulating the phosphorylation state of Mst3. To distinguish between these two possibilities, we tested whether selectively reducing the amount of PP2A associated with striatin would increase the steady-state phosphorylation level of Mst3 in those striatin complexes. We reasoned that if PP2A in the striatin complex were responsible for dephosphorylation of striatin-associated Mst3, then mutant striatins with reduced PP2A binding would bind phosphorylated Mst3 but dephosphorylate it at a reduced rate, causing the accumulation of hyperphosphorylated Mst3 in those striatin complexes.

To test this prediction, the four PP2A-deficient striatin coiled-coil domain point mutants were used. In cells expressing these mutants, PP2A that is not complexed with striatin (~98% of PP2A in the cell [8]) will be unaffected; only the amount of PP2A associated with HA-striatin will be affected. We compared the ratio of the upper (hyperphosphorylated) and lower bands of Mst3 associated with the PP2A-deficient striatin mutants with the ratio of these Mst3 bands associated with wild-type striatin. Figure 8C (HA-IP) shows the results of a representative experiment. Mst3 associated with striatin mutants deficient in binding PP2A has a greater proportion of the upper band than Mst3 associated with wild-type striatin, indicating that there is an increased amount of hyperphosphorylated Mst3 associated with the PP2A-deficient striatin mutants. Quantitative analysis of several experiments showed that Mst3 hyperphosphorylation was significantly increased in all four of our PP2A-deficient striatin complexes (Figure 8D; black columns), with the greatest increase in the R100S/R101E mutant shown in Figure 4 to be the most defective in binding PP2A. The reduction of PP2A binding to striatin causes the hyperphosphorylation (gel shift) of a large fraction of the associated Mst3, as can be seen most clearly by visual comparison of the Mst3 bands in the wild-type and R100S/R101E lanes in the HA-IP panel of Figure 8C. More than half of Mst3 associated with the R100S/R101E mutant is in the upper band.

To determine the effect on the total Mst3 population in the cell, we also analyzed the amount of hyperphosphorylation (upper band) of Mst3 in lysates from cells expressing exogenous wild-type or PP2A-deficient mutant striatins. Figure 8C-D (lysate panel and white columns) show that expression of PP2A-deficient striatin does cause an increase in total hyperphosphorylated Mst3 in cells. The most robust increase, ~2.5-fold, was again induced by expression of the striatin mutant most deficient in binding PP2A, R100S/R101E.

To definitively prove that the gel shift in Mst3 observed with PP2A-deficient striatin mutants is due to increased phosphorylation and not some other modification, we tested whether gel-shifted Mst3 associated with R100S/R101E striatin could be eliminated by treatment with purified PP2A. The slower migrating form of Mst3 disappeared when denatured R100S/R101E striatin immunoprecipitates were incubated with purified PP2A but not when they were incubated with PP2A and okadaic acid (Figure 8E). Together, these results indicate that PP2A in the striatin complex regulates the phosphorylation state of striatin-bound Mst3.

### PP2A negatively regulates the activation of striatin-associated Mst3 kinase

**Figure 9:**
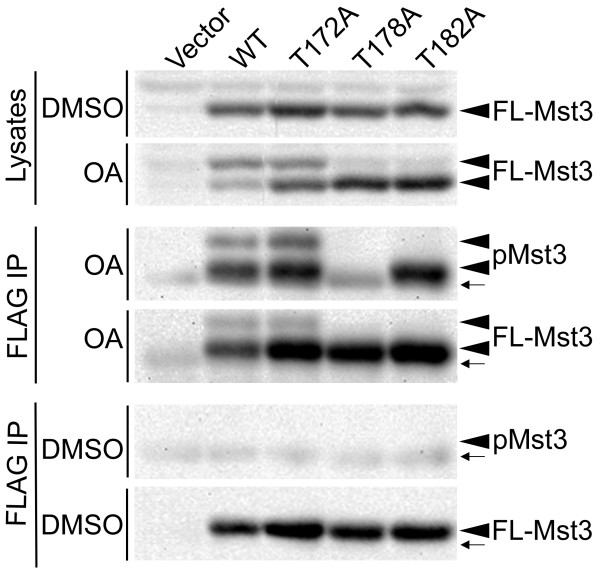
**Mutation of either threonine 178 or threonine 182 to alanine in the activation loop of Mst3 prevents the hyperphosphorylation-induced gel shift induced by okadaic acid (OA) treatment**. Three days after transfection with empty vector or FLAG epitope-tagged wild-type (WT) or mutant Mst3 isoform b-expressing plasmids, HEK293 cells were treated with either DMSO or OA (100 nM) for four hours and then lysed. Lysates were denatured as described in Methods and FLAG-Mst3 was immunoprecipitated with anti-FLAG antibody. Cell lysates (Lysates) and FLAG-Mst3 immunoprecipitates (FLAG IP) were separated by SDS-PAGE and immunoblotted with anti-FLAG antibody (FL-Mst3) and phospho-specific antibody recognizing the threonine 178 autophosphorylation site of Mst3 (pMst3). The small arrows denote the position of IgG heavy chain in the immunoprecipitates. In anti-FLAG immunoblots of lysates from DMSO-treated cells (top panel), a background band is seen at the expected position of hyperphosphorylated Mst3 in all lanes. The fact that this band is present in the vector control lysate lane and absent in anti-FLAG Mst3 immunoprecipitates prepared from these lysates (bottom panel) shows that this band is not Mst3. Okadaic acid induces the presence of the upper band of FLAG-tagged Mst3 in wild-type (WT) and T172A Mst3-expressing cells (clearly darker than the vector control background band), but not in T178A and T182A Mst3-expressing cells (second panel from the top).

To gain insight into the functional significance of the PP2A-regulated phosphorylation of Mst3 in the striatin complex, we determined the sites of phosphorylation on Mst3 necessary for the observed gel shift. To do this, the three reported activation loop phosphorylation sites of Mst3, threonine residues 172, 178, and 182 in Mst3 isoform b (PhosphoSitePlus [32]), were individually mutated to alanine to prevent their phosphorylation. After transfection of constructs expressing FLAG-tagged wild-type and mutant Mst3 isoform b proteins into HEK293 cells and treatment of the cells with okadaic acid or vehicle control (DMSO), lysates were analyzed by immunoblotting with anti-FLAG antibody (Figure 9; compare top two panels). The results show that mutation of threonine 172 causes a small reduction in the ratio of shifted to unshifted Mst3 bands while mutation of either threonine 178 or 182 to alanine abolishes the okadaic-acid induced gel shift of Mst3.

Next an autophosphorylation site-specific (pT178) antibody was used to further analyze the T172A, T178A, and T182A Mst3 mutants. Because Mst3, Mst4, and STK25 have similar sequences in their activation loops, this antibody reacts with all of these kinases when activated by autophosphorylation. To specifically examine Mst3, lysates of okadaic acid-treated cells expressing the FLAG-tagged Mst3 isoform b proteins were first denatured to disrupt complexes by heating with SDS and reducing agent as described in Methods. Then anti-FLAG immunoprecipitates of wild-type and mutant Mst3 proteins were prepared and immunoblotted with anti-phospho-Mst3 (pT178) antibody and with anti-FLAG antibody (Figure 9; two middle panels). The results confirm the specificity of the anti-phospho-Mst3 (pT178) antibody since only IgG background is seen in the T178A pMst3 immunoblot lane while a strong band of the T178A mutant protein is seen in the FLAG-Mst3 (FL-Mst3) immunoblot panel. The results also reveal that the T182A Mst3 mutant is phosphorylated robustly on threonine 178 and that Mst3 phosphorylated on threonine 178 is present in both the lower and upper bands of the Mst3 doublet in the wild-type and T172A lanes. Thus, phosphorylation of threonine 178 is not sufficient to generate the upper Mst3 band, but appears to be a prerequisite for its formation. No threonine 178 phosphorylation was detected on any of the FLAG-tagged Mst3 proteins when FLAG-Mst3 immunoprecipitates were prepared from vehicle control (DMSO)-treated cells (Figure 9; bottom two panels), indicating that the Mst3 autophosphorylation on threonine 178 seen with okadaic acid-treated cells was induced by okadaic acid and not pre-existing. Together, the results in this section show that both the upper and lower Mst3 bands contain phosphorylated Mst3 species but generation of the upper, hyperphosphorylated band appears to require at a minimum phosphorylation of both threonine 178 and threonine 182 in the activation loop of Mst3. Thus, the gel shift seen with PP2A-deficient striatin mutants is indicative of, but probably an underestimate of, activation and autophosphorylation of Mst3.

## Discussion

**Figure 10:**
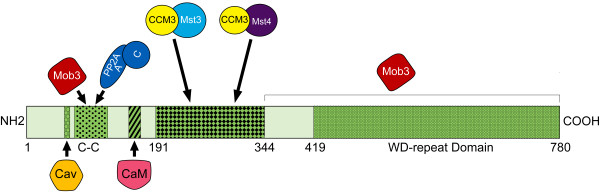
**Model of human wild-type striatin complex, based on results from previous studies and the current study**. Schematic of wild-type human striatin, drawn to scale, showing the locations of its previously published protein-interaction motifs/domains and residues or domains determined in this study to be important for binding of the striatin-associated proteins. Previously published domains: Caveolin (Cav)-binding motif (aa53-63); C-C, coiled-coil region (aa70-116); Calmodulin (CaM)-binding domain (aa149-166); WD-repeat domain (aa419-780). The Cav-binding motif and CaM-binding domain are indicated by arrows with corresponding proteins below. Estrogen receptor alpha (not shown) has been found to bind to striatin amino acids 1-203 [23]. Upper labels (from this study): Names are as indicated. Residues important for the binding of these proteins are indicated by arrows or by brackets. Mst4 binding was not affected as dramatically as Mst3 by Δ(227-309) so it may have an overlapping, but not identical, binding site. Note: The amino acid stretch in striatin important for Mst3/CCM3 association contains a small region of amino acid homology with PP2A B' B-type subunits that we previously identified [8]. Whether these residues play a role in binding Mst3/CCM3 remains to be tested.

The results of this study help define the architecture and regulation of the striatin family complexes. A model for the organization of the striatin complex is presented in Figure 10. The creation of striatin point mutants deficient in PP2A association facilitated a set of experiments clearly identifying striatin-associated PP2A as the phosphatase negatively regulating Mst3 activation in striatin family complexes. Moreover, mutational analysis of Mst3 autophosphorylation sites and studies with the PP2A inhibitor, okadaic acid, provided new insight on Mst3 activation.

The A and C subunits of PP2A were previously shown to bind to striatin family complexes but the region of striatin to which the A/C heterodimer bound was unknown [8]. Our current data show that the coiled-coil domain of striatin mediates the formation of this PP2A heterotrimer (Figure 10). Although we cannot rule out a direct contribution of the caveolin-binding motif, the fact that double or triple point mutations in the middle of the coiled-coil/oligomerization domain of striatin almost completely disrupt PP2A association suggests that the primary determinants for PP2A binding are near the middle of the coiled-coil domain (residues 84-105).

Our data also suggest that PP2A association with striatin is dependent on oligomerization of striatin complexes. Of the charged coiled-coil residues that were mutated to disrupt PP2A binding to striatin, only arginine 88 is predicted to be at the dimerization interface when either NCOILS [33] or Paircoil2 [34] prediction programs are used, while the other residues are predicted to be more available to potentially interact with PP2A. However, arginine 101, mutated in the R100S/R101E mutant, is part of a signature in striatin called a trimerization motif [35], and could have an effect on topology as well. Of the hydrophobic coiled-coil mutations, leucine 84, isoleucine 102, and leucine 105 are predicted to be at the dimerization interface, while leucine 94 may be more accessible. Thus, it is interesting that the striatin mutant L84A/L94A/L105A is significantly more defective in PP2A binding than the mutant L84A/L94A/I102A (p = 0.029), because the only difference between these two mutants is in amino acids predicted to be at the interface of the helices. These results are consistent with the idea that association of the PP2A A/C heterodimer is sensitive to local alterations in the dimerization interface of striatin. We hypothesize that this sensitivity results from PP2A binding asymmetrically across the dimerization interface to more than one striatin family coiled-coil domain. Further support for this model comes from the fact that deletion of the caveolin-binding motif, which leaves the residues mutated above intact, abolishes oligomerization of striatin and its association with PP2A. Thus, although a precise understanding of the effects of the different striatin mutants on PP2A association may await crystallization studies, our current data are consistent with a model in which the PP2A A/C heterodimer binds to the coiled-coil domain of striatin family members in an oligomerization-dependent manner. Because we have shown that Mst3 binding to striatin is not dependent on striatin family oligomerization, this model provides an attractive feature of restricting the regulation of striatin-associated Mst3 by PP2A until oligomerization occurs.

A surprising result from this study was the complete loss of oligomerization caused by deletion of the caveolin-binding motif within striatin (mutant Δ(53-66)). A previous study [15] reported that a fusion protein consisting of residues 81-131 of mouse SG2NA (corresponding to striatin amino acids 65-115) fused to the C-terminus of GFP did not oligomerize for unknown reasons. One possible explanation for both of these results is that striatin family oligomerization requires binding of caveolin. This would have to be a transient requirement, however, because we could find no associated caveolin even in wild-type striatin immunoprecipitates (data not shown) and no caveolin was found in striatin family complexes in a recent proteomics study [9]. Alternatively, loss of oligomerization could result from one or more of the deleted residues being directly required for oligomerization. Analyses using NCOILS [33] and Paircoil2 [34] prediction programs suggest that the N- and C-terminal limits previously assigned to the coiled-coil domain of striatin (70-116; [11]) may need to be extended. Using a 21 residue window with NCOILS, which is recommended for locating the ends of coil-coils, and an unweighted analysis (heptad residues a-g given equal weight), probabilities of almost 1 are obtained for residues 61-69 being part of the coiled-coil of human striatin. Weighted analysis (residues a and d given 2.5-fold more weight than the other heptad residues) using the same window still assigns these residues high probability (0.621 for residues 61-63 and 0.956 for residues 64-69) of being part of the coiled-coil. Results from similar analyses of human SG2NA suggest that SG2NA residues corresponding to striatin aa61-69 (unweighted) or aa64-69 (weighted) have a high probability of being part of the coiled-coil. Finally, for human zinedin, NCOILS predicts unweighted and weighted N-terminal ends corresponding to striatin aa56 and aa61, respectively. For striatin, SG2NA, and zinedin, these analyses also suggest that the C-terminal end of the coiled-coil may be around aa120-121. Paircoil2 analysis of these proteins predicts a similar end to the coiled-coil but an even earlier start to it, ranging from striatin aa56-61, potentially including much of the caveolin-binding motif, residues 53-63. The results of these analyses are consistent with the idea that our caveolin deletion mutant and the GFP-SG2NA fusion protein published by others may have removed amino acids critical to the formation of striatin coiled-coils. Since the missing residues common to both of these constructs are residues 53-64, it is possible that these are the critical amino acids. In fact, the complete loss of coiled-coil formation caused by our small caveolin-binding motif deletion is reminiscent of "trigger" sequences, sequences absolutely required for coiled-coil formation [36]. Additional analyses will be needed to firmly define the striatin coiled-coil and resolve these possibilities.

There are at least two distinct domains within striatin (aa1-269 and aa310-780) that interact directly or indirectly with Mob (Figure 10). The C-terminal striatin residues important for Mob3 binding were further narrowed down by the fact that deletion of striatin residues 270-344 had no significant effect on Mob3 binding (Figure 5C). This result suggested that the most important C-terminal determinants of striatin for Mob binding likely occur after residue 344 and include the WD domain. However, because of the larger standard deviation of Mob3 binding to the Δ(270-344) mutant, a contribution of striatin residues 310-344 cannot be completely ruled out. In the N-terminus of striatin, Mob3 binding seems to be largely restricted to the coiled-coil domain because deletion mutants collectively spanning residues 115-269 and a mutant deleting the caveolin-binding motif had no significant effect on Mob3 binding, while deletion of the coiled-coil domain had the same reduction of Mob3 binding as the Δ(3-309) striatin mutant. The fact that Mob3 binds much better to the caveolin-binding motif deletion mutant than to the coiled-coil deletion mutant and close to wild-type levels also supports the idea that Mob3 binding to the striatin N-terminus is not highly dependent on oligomerization. This result suggests that Mob3 binding to the striatin N-terminus is not solely due to indirect binding of Mob3 through oligomerization of endogenous striatin family members with mutants. However, additional studies are necessary to strengthen this conclusion. It has not been firmly established whether Mob3 binds directly or indirectly to striatin. A previous study identified Mob3 (phocein) by its binding to striatin in a two-hybrid assay but no additional data was presented to rule out the possibility that the interaction was mediated by a conserved protein in yeast (e.g., PP2A A and C subunits) bridging the association. Mob3 binds striatin independently of PP2A C subunit, Mst3, and CCM3 because when close to 85% of the latter associations were disrupted by various mutations little to no reduction in Mob3 binding was seen. Since PP2A C subunit, Mst3, and CCM3 are other core striatin binding partners, the results support the model that Mob3 binds directly to striatin. However, the question of whether striatin binds two molecules of Mob3 or uses two binding domains to interact with a single molecule of Mob3 still needs to be resolved. Given our other data on the organization of the striatin complex, it is tempting to speculate that binding of two striatin domains to one Mob3 molecule might function in part to bring Mst3 and PP2A into proximity in the 3D structure of striatin.

The results of this study show that striatin-associated PP2A is the phosphatase responsible for negatively regulating the phosphorylation and activation of Mst3 in striatin family complexes. Four separate striatin point mutants deficient in PP2A binding showed hyperphosphorylation of associated Mst3. Moreover, hyperphosphorylated, gel-shifted Mst3 could also be detected in lysates from cells expressing these mutants, indicating that these mutants cause the hyperphosphorylation of a significant portion of the Mst3 in the cell. Using Mst3 activation loop point mutants, autophosphorylation site T178-specific antibody, and okadaic acid to inhibit PP2A and activate Mst3 in vivo [37], we demonstrated that gel-shifted Mst3 was indicative of Mst3 activation and autophosphorylation in vivo. This conclusion is further supported by previous studies that reported a gel shift of Mst3 upon autophosphorylation in vitro [19,38], and a consequential increase in kinase activity after autophosphorylation [38]. Our data further show that the Mst3 gel shift represents an underestimation of the amount of Mst3 autophosphorylated on threonine 178, the reported site of Mst3 autophosphorylation for Mst3b [19], because phosphorylation at both threonines 178 and 182 on Mst3b appears to be required to obtain the observed gel shift. Thus, striatin-associated PP2A negatively regulates the phosphorylation and activation of Mst3, and likely of the other related kinases in striatin family complexes, Mst4 (which is also gel-shifted upon okadaic acid treatment) and STK25, as well. The fact that reduction in striatin-associated PP2A is sufficient to cause activation of Mst3 suggests that one way the Mst3 and the other GCKIII kinases could be regulated is by modulation of striatin-associated PP2A activity or modulation of PP2A's access to Mst3 in striatin family complexes. An important goal of future research on these complexes will be to determine if these mechanisms exist, and if so, to elucidate them.

Previous data suggested that threonine 182 was not a major autophosphorylation site on Mst3 [19]. However, this data was largely based on peptide phosphorylation studies. We (Figure 8A) and others [19,38] have found that incubation of immunoprecipitated Mst3 in the presence of ATP and manganese generates the hypershifted form of Mst3 in vitro. Considered together with the current result that mutation of threonine 182 to alanine completely prevents the gel-shift of Mst3 without blocking the phosphorylation of threonine 178, this finding supports the notion that threonine 182 is a major autophosphorylation site of Mst3. The fact that there is no detectable gel shift when either threonine 178 or threonine 182 is mutated to alanine is intriguing. One possible explanation is that simultaneous phosphorylation at both of these sites is required to produce the gel shift. Another possibility is that there may be a preference for sequential phosphorylation of Mst3 activation loop phosphorylation sites, with phosphorylation at threonine 178 being a prerequisite for phosphorylation at threonine 182, which in turn is required for the observed gel shift to occur. Evidence for an interdependence in activation loop phosphorylations has been noted previously for other kinases such as Chk2 [39]. Availability of a phospho-specific antibody for threonine 182 of Mst3 would be helpful for distinguishing between these and other possibilities.

Deletion of the calmodulin-binding domain (Δ(148-166) striatin), which has no effect on striatin oligomerization or binding of Mob3 to striatin and little effect on PP2A association with striatin (Figure 3C-D), caused a 3- to 4-fold increase in both Mst3 and Mst4 association with striatin. This result is consistent with the possibility that the calmodulin-binding domain negatively regulates the binding of Mst3 and Mst4 in striatin family complexes. The effect of this deletion mutant is not simply due to the removal of amino acids in this general area of the striatin sequence because adjacent deletions on either side of the calmodulin-binding domain had either no effect or only a modest 25% increase in Mst3 binding. Given that we and others have shown that striatin family members associate with calmodulin in a calcium-dependent manner [8,11,12], calcium may regulate Mst3 and Mst4 recruitment to striatin complexes by regulating calmodulin binding to striatin. However, it is also possible that removal of these specific sequences modifies Mst3 binding in a manner that will not be recapitulated by changes in calcium. Further experimentation will be required to distinguish between these possibilities.

## Conclusions

The results of this study help define the architecture of striatin complexes by identifying residues of striatin important for complex formation with Mob3, PP2A, Mst3, Mst4, and CCM3. Our data support the novel hypothesis that PP2A binding to the coiled-coil domain requires striatin family oligomerization, which has implications for the assembly of striatin family complexes and the ability of PP2A to regulate other components of these complexes. The finding of residues critical for striatin oligomerization N-terminal to its previously assigned coiled-coil domain combined with results from analyses using coiled-coil prediction programs suggest that striatin's coiled-coil domain may begin earlier in the striatin sequence than previously thought. In addition, our results support a model in which striatin binds Mst3 and CCM3 likely as a dimer via residues lying between striatin's calmodulin-binding and WD-domains and recruits the PP2A A/C heterodimer to its coiled-coil/oligomerization domain to regulate Mst3. Striatin-associated PP2A is critical for efficient dephosphorylation and inactivation of striatin-associated Mst3. Upon inhibition of PP2A by okadaic acid, Mst3 activation appears to involve autophosphorylation of its activation loop phosphorylation sites, resulting in a gel shift on SDS-PAGE. Mob3 can associate with striatin sequences C-terminal to the Mst3 binding site but also with sequences proximal to striatin-associated PP2A, consistent with a possible role for Mob 3 in the regulation of Mst3 by PP2A.

## Methods

### Antibodies

Anti- HA-tag antibodies, 12CA5 and F7 (available from Santa Cruz Biotechnology) were used for immunoprecipitation of HA-tagged proteins, while anti-HA antibody 16b12 (Covance) was used for immunoblotting. Other antibodies used include mouse monoclonal anti-FLAG epitope tag antibody M2 (Sigma), PP2A C subunit antibody (BD Transduction Labs), anti-SG2NA mouse monoclonal antibody S68 [14] (available from Millipore and Santa Cruz Biotechnology or by request to the corresponding author), anti-Mob3 rabbit polyclonal antibody RK130 [14], and rabbit monoclonal antibodies anti-Mst3, anti-Mst4, and anti-Mst4(pT178)/Mst3(pT190)/STK25 (pT174) (Epitomics, Inc.). Mst3 has two isoforms, a longer isoform, referred to as isoform a, and a shorter isoform, isoform b. The anti-phospho Mst4/Mst3/STK25 antibody mentioned above detects Mst3 isoform a that is phosphorylated on threonine 190 in its activation loop or Mst3 isoform b phosphorylated on its corresponding activation loop threonine, threonine 178. Because we used FLAG epitope-tagged Mst3 isoform b for experiments utilizing this antibody, we have referred to this antibody as anti-Mst3 (pT178) in this report.

### Plasmids, mutagenesis, and creation of stable cell lines

A human striatin cDNA clone was assembled from ESTs by standard approaches, sequenced in its entirety to confirm that it contained no mutations, and inserted into pEGFP-N3 with a modified multiple cloning site to make pEGFP-N3-wild-type striatin. Standard restriction cloning and PCR mutagenesis techniques were then used to make point mutations or small deletions in pEGFP-N3-wild-type striatin, except that in most cases PCR was carried out using Herculase^® ^II polymerase (Stratagene) because this enzyme was necessary for efficient PCR of GC-rich striatin sequences. The striatin deletion mutants containing only the coiled-coil domain or coiled-coil and caveolin-binding domains were made by standard cloning procedures in a modified lentivirus plasmid (pLenti6/V5-D-Topo; Invitrogen) with an N-terminal double HA epitope tag, but were used as plasmids (i.e., transfected). The empty vector and wild-type striatin expressed in the same construct as the mutants were used as controls. All striatin mutants were sequenced.

The cDNA of human Mst3 isoform b (Mst3b; Clone ID HsCD00042929) was obtained from the Dana-Farber/Harvard Cancer Center DNA Resource Core. The hMst3b ORF was PCR-amplified and ligated into HindIII-EcoRI sites of pcDNA3.1 with a HindIII-HindIII N-terminal FLAG epitope tag, excised with the tag by cutting with NheI and ApaI, and ligated into an XbaI-ApaI digested backbone from the plasmid pLenti6/V5-D-Topo. Standard PCR mutagenesis procedures were used to introduce the threonine to alanine point mutations in the activation loop. The cDNA of human CCM3 (Catalog No. SC320246) was obtained from OriGene Technologies, Inc. and the hCCM3 ORF was PCR-amplified and inserted into a modified pLenti6/V5-D-Topo plasmid with an N-terminal FLAG epitope tag. To create HEK293 cells stably expressing FLAG-tagged hCCM3, cells were infected with lentiviruses that express either empty lentivirus plasmid with a FLAG epitope or lentivirus plasmid expressing N-terminally FLAG-tagged hCCM3. Infected cells were incubated in media supplemented with 7 ug/ml of blasticidin to select cells that stably express FLAG-tagged CCM3. The selected stable cell lines were used to perform co-immunoprecipitation experiments after transfecting with a set of striatin deletion mutants and controls.

### Cell culture, transfections, and cell lysis

HEK293 cells were cultured in Dulbecco's modified Eagle's medium supplemented with 5 or 10% fetal calf serum at 37°C in 10% CO_2_. For experiments, HEK293 cells were plated into 60 mm dishes at 15% confluence and grown at 37°C for 16-24 hours. The cells were then transfected using FuGENE™ 6 transfection reagent (Roche Diagnostics) according to the manufacturer's protocol. Control inclusion of a small amount (one-tenth of other DNAs) of GFP expressing plasmid in some experiments showed > 50% of cells were routinely being transfected. After a minimum of 48 h to allow the wild-type and mutant striatins to express and bind associated proteins in the cells, the cells were washed with ice cold phosphate-buffered saline and then with IP wash buffer (0.135 M NaCl, 10% glycerol, 20 mM Tris, pH 8.0) prior to being lysed with IP lysis buffer (IP wash buffer containing 1% Nonidet P-40, 1 mM phenylmethylsufonyl fluoride, 0.04 trypsin inhibitor units/ml aprotinin, and in the case of phosphorylation-related experiments, 50 mM sodium fluoride and 100 nM okadaic acid (LC Laboratories) to prevent dephosphorylation in lysates) by rocking for 20 minutes at 4°C. Lysates were cleared by centrifugation at 13,000 × g.

### Immunoprecipitation, gel electrophoresis, and immunoblotting

To immunoprecipitate HA-tagged proteins and complexes, cleared lysates were incubated with 12CA5 anti-HA antibody and protein A-Sepharose 4B beads (Invitrogen) or F7 anti-HA-agarose conjugate (when probing for Mst3, Mst4, or pMst3; Santa Cruz Biotechnology # sc-7392 AC) for 1.5 hours at 4°C with rocking. FLAG epitope-tag immunoprecipitates were prepared in the same manner with M2 anti-FLAG antibody (Sigma-Aldrich) and recombinant protein G-Sepharose 4B beads (Invitrogen). Immune complexes were harvested by centrifugation, washed twice in phosphate buffered saline, twice in IP lysis buffer, and heated at near boiling in sample buffer for 5 minutes. Immune complexes were resolved by SDS-PAGE. Proteins were transferred to nitrocellulose for immunoblotting. Clean Blot (Pierce; 1:1000) was used for detection of Mst3, Mst4, or pMst3 in most cases to reduce background from the immunoprecipitating IgG heavy chain. Bands from immunoblotting were visualized by enhanced chemiluminescence and a Fluor S-Max Chemilumimager (Bio-Rad). The chemilumimager directly measures band intensities without the use of film via a supercooled CCD camera that provides linear data over 4.8 orders of magnitude. This method yields highly reproducible results that do not vary with image capture times. For mapping experiments in which the amount of associated proteins were being compared, only experiments in which similar expression of wild-type and mutants were obtained were used for quantitation to avoid an artificial bias from different levels of expression. Quantitation of striatin levels in vector control cells and HA-wild-type striatin transfected cells indicates that 1 ug striatin transfected into a 60 mm dish of cells results in close to a 1:1 level of expression of HA-wild-type striatin to endogenous striatin (data not shown). The amount of DNA used for experiments in this study ranged from 0.5-2 ug.

### Phosphorylation of Mst3 in vitro

Three days after transfection of HEK293 cells with a plasmid encoding FLAG-tagged wild-type Mst3, FLAG immunoprecipitates were prepared and washed twice with phosphate buffered saline (pH 7.3), twice with lysis buffer, and once with tris buffered saline. The immune complexes were combined, divided equally into three tubes, and then suspended in a buffer containing 40 mM 1,4 Piperazine-diethanesulfonic acid (pH7.0) and 2 mM MnCl_2_. Two samples were incubated with DMSO (vehicle control) while the other sample was incubated with 1 μM staurosporine, all for 30 min at room temperature. Then ATP was added to a final concentration of 20 μM to one vehicle control aliquot and to the staurosporine-containing aliquot. After incubation for 30 min at room temperature all three reactions were analyzed by SDS-PAGE and Mst3 protein bands detected by immunoblotting.

### Dephosphorylation of Mst3 in vitro

Three days after transfection with HA-tagged R100S/R101E striatin, HEK293 cells were lysed in the absence of phosphatase inhibitors and HA-striatin immune complexes were prepared. The immune complexes were denatured by heating at near boiling for 5 minutes in lysis buffer containing 0.5% SDS and 5 mM β-mercaptoethanol, centrifuged to remove the protein A sepharose beads, and then the supernatant was divided into three equal portions. Each sample was then diluted with a four-fold excess of lysis buffer to capture excess SDS into mixed micelles with Nonidet P-40. For two of the samples, the lysis buffer contained purified PP2A (Millipore) plus DMSO (vehicle control) or 100 nM okadaic acid. After incubation for 60 minutes at 30°C, the samples were analyzed by SDS-PAGE and Mst3 protein bands were detected by immunoblotting.

### Immunoprecipitation of denatured lysates

To denature lysates prior to immunoprecipitation, cells were first lysed in lysis buffer as described above and then, after clearing the lysates, SDS and β-mercaptoethanol were added to final concentrations of 0.5% and 5 mM, respectively. The lysates were then heated at near boiling for 5 minutes, diluted 1:4 with IP lysis buffer to capture excess SDS into mixed micelles, and then immunoprecipitated as described above.

## Competing interests

Dr. David Pallas is entitled to royalty from the sale of products related to the research described in this paper by Millipore Inc., Santa Cruz Biotechnologies Inc., Invitrogen Corp., and Novus Biologicals Inc. In addition, this same author serves as a consultant to Millipore. The terms of these arrangements have been reviewed and approved by Emory University in accordance with its conflict of interest policies.

## Authors' contributions

JG and JH carried out most of the study, contributed ideas towards its design and execution, and contributed to the preparation of the manuscript. KC, CAJ, QLK, and CSM participated in execution, analysis, and troubleshooting of the striatin structure-function experiments. CSM and RHK contributed ideas to the study and participated in the drafting of the manuscript. DCP conceived of the study, and participated in its design, coordination and execution, and helped draft the manuscript. All authors read and approved the final manuscript.
